# Caries and edentulism trends among Brazilian older adults: a comparative analysis of 2003, 2010, and 2023 surveys

**DOI:** 10.1590/1807-3107bor-2025.vol39.0050

**Published:** 2025-05-19

**Authors:** Raquel Conceição FERREIRA, Andréa Maria Duarte VARGAS, Rosa Núbia Vieira de MOURA, Maria Luíza Viana FONSECA, Viviane Elisângela GOMES, Elisa Lopes PINHEIRO, Sandra Cecília Aires CARTAXO, Rafaela da Silveira PINTO

**Affiliations:** (a) Universidade Federal de Minas Gerais – UFMG, School of Dentistry, Department of Social and Preventive Dentistry, Belo Horizonte, MG, Brazil.; (b) Ministério da Saúde, National Oral Health Coordination, Brasília, DF, Brazil.

**Keywords:** Oral Health, Aged, Health Surveys, Tooth Loss, Dental Caries, Health Inequities

## Abstract

This study compared the experience of dental caries and the prevalence of edentulism in 2003, 2010, and 2023 for individuals aged 65 to 74 in Brazil by region and according to self-declared race/skin color and years of schooling. A probabilistic cluster sample obtained from three national oral health surveys was analyzed. Information from oral health examinations for dental caries, according to the World Health Organization, common to three surveys, were used. The statistical significance of differences between estimates from each survey was evaluated for a linear combination of coefficients after mean or proportion estimation command by subpopulations and two-sided t-tests. Poisson and logistic regression models were employed to estimate changes between surveys while controlling for sociodemographic characteristics. The sampling design and sample weight were considered for the estimates. The analysis of data from 5,349 (2003), 7,509 (2010), and 9,745 (2023) individuals revealed a significant reduction in the DMFT index from 27.60 (2003) and 27.53 (2010) to 23.55 (2023) and in the prevalence of edentulism from 53.34% (2003) and 53.38% (2010) to 36.32% (2023). The number of missing teeth decreased by 14.46% between 2023 and 2010, with the greatest variation among white individuals and those with higher educational levels. The number of filled teeth increased. Adjusted regression models confirmed significant reductions in the DMFT index and the prevalence of edentulism between 2023 and 2003. The elderly Brazilian population is retaining more natural teeth. However, reductions in tooth loss were unequal, occurring primarily among white individuals and those with higher educational levels.

## Introduction

Oral diseases are among the most prevalent non-communicable chronic diseases worldwide despite being largely preventable.^1^ Their high prevalence highlights significant social and economic inequalities and insufficient investment in prevention and treatment, especially in low- and middle-income countries.^1^ These diseases arise from a complex interaction of biological, behavioral, social, and economic factors influenced by living conditions and access to healthcare services. Tooth loss, a direct consequence of prolonged exposure to oral diseases and their treatment, exemplifies these broader issues.^2,3^ It also reflects patient and dentist attitudes, the dynamics of the dentist-patient relationship, the accessibility of dental services, and prevailing philosophies of dental care.^4^

Edentulism, defined as the complete loss of natural teeth, profoundly affects physical and mental health. It impairs chewing ability, limits food choices, alters nutritional intake, contributes to weight loss, increases the risk of systemic diseases, and reduces the quality of life.^5,6^ Aging is a significant risk factor, with edentulism being more prevalent among older adults, particularly those aged 60 and above, where severe tooth loss peaks.^2^

The World Health Organization (WHO) identifies dental caries and periodontal disease as the primary causes of tooth loss, underscoring their importance as public health concerns.^7^ These conditions significantly contribute to tooth extractions.^8-10^ The declining prevalence of dental caries in many populations has correlated with reduced tooth loss and edentulism. Notably, many developed countries report decreasing tooth loss across all age groups.^9,11-13^

In Brazil, oral health indicators are systematically monitored through National Oral Health Surveys (SB Brasil) as a National Oral Health Policy (PNSB, in Portuguese) surveillance strategy. Dental caries is assessed using the WHO-recommended Decayed, Missing, and Filled Teeth (DMFT) index, which measures cumulative caries experience. Separating the DMFT components—decayed, missing, and filled teeth—offers critical insights into trends and shifts in caries distribution, particularly among older adults. This detailed analysis informs targeted interventions and public health strategies.

As key oral health indicators, caries and edentulism are closely linked to social determinants. Poor oral health disproportionately affects socioeconomically marginalized groups.^1,14,15^ Poverty, limited education, and other adverse socioeconomic conditions exacerbate oral health disparities, as demonstrated in multiple countries.^7^ The persistence of these disparities, even in nations with overall better health outcomes, remains a major global challenge.^1^ In Brazil, the PNSB, implemented in 2004, aimed to reduce oral health inequalities, with equity and universality as guiding principles. It was oriented by the principles of health promotion, seeking to expand access to preventive and conservative treatments while moving beyond the historically mutilative care model that prevailed in emergency settings.

Therefore, monitoring these indicators should account for their unequal distribution among population subgroups, particularly those defined by socioeconomic status. The aim was to compare caries experience and the prevalence of edentulism among elderly individuals aged 65 to 74 years across three national epidemiological surveys conducted in Brazil in 2003, 2010, and 2023, stratified by Brazilian regions, and according to years of schooling and self-reported race/skin color.

## Methods

This study was conducted using cross-sectional data from the national population-based oral health surveys of 2003, 2010, and 2023, all coordinated by the Brazilian Ministry of Health.

The sampling plans for the SB Brasil surveys from 2003, 2010, and 2023 involved multi-stage cluster sampling across different geographical domains. In 2003, the survey covered 250 municipalities across five macro-regions, with participants selected from households in census tracts. For 2010, the sampling expanded to 32 domains, including state capitals and interior municipalities, with two or three stages of selection. The sample size was 250 per domain, with participants in specific age groups selected through cluster sampling. In 2023, the survey covered 53 domains, including all states and capitals, with a sample size of 300 for capitals and 100 for the interior per state, totaling 400 samples per domain. The sampling design allowed for estimates to be made for each of the five Brazilian regions. More details on the sampling plan for the three surveys have been described in previous publications.^16-18^ This study analyzed data from the 65-74 age group.

### Data Collection

Data collection in all three surveys involved interviews and oral examinations conducted by healthcare professionals from the Brazilian Unified Health System (SUS), including a dental surgeon and a dental health technician or assistant. Field teams were trained to ensure consistency, with examiners undergoing extensive theoretical and practical training, achieving high agreement coefficients (Kappa ≥ 0.65 in 2003 and 2010, ≥ 0.61 in 2023). In 2003 and 2010, training included in-person workshops with practical exercises on oral health conditions and calibration. In 2023, due to the COVID-19 pandemic, training was conducted online, focusing on methodology, oral health codes, and calibration using photographs.^19,20^ The methods used were described in detail in previous publications.^17,18,20,21^

### Outcomes

The outcomes were the dental caries experience and edentulism. The three surveys used the WHO codes and criteria to assess dental caries in the crowns of permanent teeth.^22^ Caries was defined as the presence of a lesion in a pit or fissure, or on a smooth tooth surface, or an unmistakable cavity, undermined enamel, or a detectably softened floor or wall, or the presence of a temporary filling (excluding glass ionomer restorations). Restoration with caries was observed when one or more permanent restorations and one or more carious areas were present simultaneously, regardless of whether the caries lesions were primary or secondary. When one or more definitive restorations were observed without primary or secondary caries, the tooth was classified as filled without caries. Based on this evaluation, the DMFT index (number of permanent teeth decayed, missing, and filled) was calculated, as recommended by the WHO.^22^ The total number of decayed teeth was calculated by summing decayed teeth and filled teeth with caries. Additionally, the total number of filled teeth and the total number of missing teeth were also calculated. The means for the DMFT and each condition—decayed, filled, and missing teeth—were obtained. Edentulism was defined as losing all teeth, including teeth lost for other reasons.

### Covariates

The covariates on the socio-demographic profile (age, sex, self-reported race/skin color, and education level of participants), which were collected using the same methodology across the three surveys, were analyzed. Ages ranged from 64 to 74 years and were categorized into 64 to 70 and 71 to 74 years. Self-reported race/skin color was assessed according to the Brazilian Institute of Geography and Statistics (in Portuguese, IBGE) methodology as white, black, Asian, mixed-race, and Indigenous. Years of schooling were evaluated based on the highest grade (level) completed by the participant. This was converted into years of study without failing and categorized as 0 (did not study), 1 to 4, 5 to 8, 9 to 11, and 12 or more years of schooling.

### Data Analysis

The databases from the three surveys were merged, adjustments were made to the codes of the common variables, and a new variable “year” was added to identify each survey. All databases included weighting variables. For the 2003 survey, the sample weights were calculated by Queiroz et al.^23^ Estimates of means, percentages, and their 95% confidence intervals were calculated considering the sample design variables and applying the weights derived from the sampling process for the three surveys using the svy command in Stata v. 18 (StataCorp LP, College Station, USA). The same estimates were obtained considering the total sample, analyzing data by Brazil and by regions. Additionally, estimates were obtained for subgroups defined by sex, age group, self-reported race/skin color, and years of schooling. The observed change in the means of the DMFT index and its components, as well as the prevalence of edentulism between surveys, was calculated: 2023 compared to 2010 and 2003, and 2010 compared to 2003. This change was presented as Δ2023_2010, Δ2023_2003, and Δ2010_2003. The significance of the change was tested by hypothesis testing, dividing Δ by the standard error of the change and estimating the confidence interval of the change at a 95% significance level, rejecting the null hypothesis when the confidence interval did not include zero. Differences in means between the years were estimated using the lincom command in Stata®, considering subpopulations defined by the variables of interest: region, sex, age group, self-reported race/skin color, and years of schooling. Assuming that the effects of sex, age group, self-reported race/skin color, and years of schooling were constant across the three surveys, the change estimate between surveys was obtained by controlling for these factors using a regression model. A Poisson regression model was adjusted to assess changes in the DMFT index and the number of decayed, missing, and filled teeth, including “survey year” as a covariate, according to the methodological guidance of Lee et al., 2007.^24^ The same strategy was applied using a logistic regression model for edentulism. The models were adjusted for sex, age group, self-reported race/skin color, and years of schooling.

The National Research Ethics Committee approved the three surveys (2003: approval protocol number: 1356; 2010: approval protocol number: 15.498; 2023: approval protocol number: 4.823.054), and the participants signed an informed consent form

## Results

In 2003, 5,349 elderly individuals were interviewed and examined. The sample sizes in 2010 and 2023 were 7,509 (93.8%) and 9,745 (91.10%). The gender distribution was similar in all three years, with a higher percentage of women, 2003: 56.2%, 2010: 61.8%, and 2023: 60.4%. Participants who self-identified as white were the majority in all three surveys (2003: 52.8%, 2010: 54.6%, and 2023: 49.6%), followed by mixed-race (2003: 33.23%, 2010: 29.8%, and 2023: 36.1%), black (2003: 10.0%, 2010: 13.7%, and 2023: 12.8%), and Asian or Indigenous (2003: 3.9%, 2010: 1.8%, and 2023: 1.5%). The distribution related to educational levels across the years was: illiterate (2003: 28.9%, 2010: 14.8%, and 2023: 11.5%), 1 to 4 years of schooling (2003: 49.1%, 2010: 49.1%, and 2023: 27.9%), 5 to 8 years (2003: 13.6%, 2010: 20.4%, and 2023: 26.2%), 9 to 11 years (2003: 5.9%, 2010: 8.9%, and 2023: 19.1%), and 12 or more years (2003: 2.3%, 2010: 6.8%, and 2023: 15.3%).

**Figure 1:**
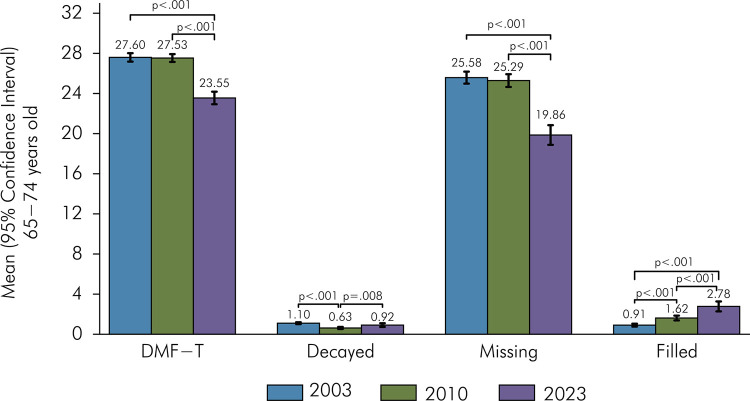
Mean of DMFT index and of decayed, filled and missing teeth among older adults in 2003, 2010 and 2023.

**Figure 2:**
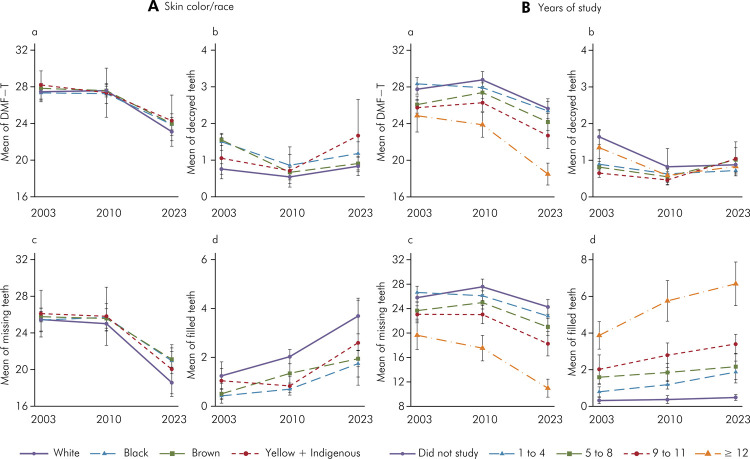
Mean DMF-T (a), decayed (b), missing (c) and filled (d) teeth by skin color/race (A) and years of study (B) in 2003, 2010 and 2023

The average DMFT index was 27.60 in 2003, 27.53 in 2010, and 23.55 in 2023 (Figure 1). A significant reduction was observed in the 2023 compared to 2003 and 2010, both for Brazil and its regions. Between 2023 and 2010, the index average decreased by 3.98 teeth, representing a percentage variation of 14.46%. Across regions, the percentage reductions in DMFT ranged from 11.06% in the Central-West to 16.93% in the Southeast. No significant changes were observed in the DMFT index between 2003 and 2010 for Brazil or the regions. Between 2023 and 2003, and between 2023 and 2010, the DMFT index decreased for most analyzed subgroups, except for participants who self-identified as Asian and Indigenous (Table 1). The largest variation between 2023 and 2010 was observed in elderly participants aged 64 to 70 (∆ 2023_2010 = -4.63), white individuals (∆ 2023_2010 = -4.48), and those with 12 or more years of schooling (∆ 2023_2010 = -5.37) (Table 1; Figure 2A and Figure 2B).

p < 0.05 indicates statistically significant changes when comparing surveys.

**Table 1:** Mean DMFT in 2003, 2010 and 2023 for Brazil, regions and subgroups according to sex, age group, self-reported race/skin color, and the estimate change and 95% confidence interval for the changes observed in the surveys.

Variables	2003	2010	2023		∆2010–2003	∆2023–2003	∆2023–2010
Brazil	27.60 (27.18–28.02)	27.53 (27.14–27.92)		23.55 (22.93–24.18)	-0.06 (-0.64–0.51)	-4.04 (-4.80; -3.29)	-3.98 (-4.72; -3.25)
Region							
North	28.61 (28.03–29.18)	28.26 (27.79–28.73)		24.29 (22.76–25.82)	-0.35 (-1.09–0.40)	-4.32 (-5.95; -2.68)	-3.97 (-5.57; -2.37)
Northeast	27.12 (26.91–27.33)	27.20 (26.89–27.50)		23.91 (22.88–24.94)	0.08 (-0.29–0.44)	-3.21 (-4.26; -2.16)	-3.29 (-4.36; -2.21)
Southeast	27.89 (27.02–28.77)	27.65 (27.04–28.25)		22.97 (21.81–24.12)	-0.25 (-1.31–0.81)	-4.93 (-6.37; -3.48)	-4.68 (-5.97; -3.38)
South	27.27 (26.91–27.62)	27.10 (26.51–27.69)		24.09 (23.17–25.01)	-0.16 (-0.86–0.53)	-3.18 (-4.17; -2.19)	-3.02 (-4.11; -1.92)
Central-West	27.56 (27.01–28.11)	27.49 (27.08–27.90)		24.45 (23.26–25.63)	-0.07 (-0.76–0.61)	-3.11 (-4.42; -1.81)	-3.04 (-4.30; -1.79
Age (years)							
64–70	26.97 (26.63–27.31)	27.34 (26.87–27.82)		22.71 (21.93–23.49)	0.37 (-0.21–0.95)	**-4.26 (-5.11; -3.41)**	**-4.63 (-5.54; -3.72)**
71–74	28.77 (27.93–29.62)	27.86 (27.34–28.37)		25.13 (24.42–25.83)	-0.91 (-1.89–0.07)	**-3.64 (-4.74; -2.55)**	**-2.73 (-3.59; -1.87)**
Sex							
Male	26.14 (25.74–26.53)	26.66 (26.07–27.26)		22.64 (21.73–23.56)	0.53 (-0.19–1.24)	**-3.49 (-4.49; -2.49)**	**-4.02 (-5.11; -2.93)**
Female	28.74 (28.19–29.28)	28.07 (27.55–28.59)		24.15 (23.51–24.79)	-0.66 (-1.42–0.08)	**-4.59 (-5.43; -3.74)**	**-3.92 (-4.75; -3.09)**
Race/skin color							
White	27.46 (25.56–28.37)	27.58 (27.09–28.07)		23.10 (22.11–24.10)	0.12 (-0.90–1.15)	**-4.35 (-5.70; -3.01)**	**-4.48 (-5.58; -3.37)**
Black	27.33 (26.36–28.31)	27.25 (26.18–28.33)		23.88 (22.71–25.05)	-0.08 (-1.53–1.36)	**-3.45 (-4.97; -1.93)**	**-3.37 (-4.95; -1.78)**
Mixed-race	27.83 (27.44–28.22)	27.59 (26.89–28.28)		23.98 (23.20–24.65)	-0.24 (-1.03–0.55)	**-3.86 (-4.63; -3.08)**	**-3.61 (-4.57; -2.65)**
Asian + Indigenous	28.20 (26.66–29.74)	27.36 (24.68–30.04)		24.30(21.51–27.10)	-0.84 (-3.93–2.25)	**-3.89 (-7.08; -0.71)**	**-3.05 (-6.92; 0.82)**
Years of schooling							
Never been to school	27.75 (27.22–28.29)	28.76 (27.85–29.68)		25.64 (24.58–26.71)	1.01 (-0.05–2.07)	**-2.11 (-3.30; -0.92)**	**-3.12 (-4.52; -1.72)**
1–4	28.34 (27.65–29.02)	27.92 (27.41–28.44)		25.38 (24.37–26.93)	-0.41 (-1.27–0.44)	**-2.95 (-4.17; -1.73)**	**-2.54 (-3.67; -1.41)**
5–8	26.07 (25.15–27.00)	27.39 (26.71–28.06)		24.17 (23.23–25.11)	1.31 (0.16–2.46)	**-1.90 (-3.22; -0.59)**	**-3.22 (-4.37; -2.06)**
9–11	25.74 (24.92–26.55)	26.27 (25.34–27.20)		22.69 (21.27–24.11)	0.54 (-0.69–1.78)	**-3.04 (-4.68; -1.41)**	**-3.58 (-5.28; -1.89)**
> 12	24.86 (23.08–26.64)	23.87 (22.51–25.23)		18.49 (17.30–19.69)	-0.98 (-3.22–1.25)	**-6.36 (-8.50; -4.22)**	**-5.37 (-7.18; -3.54)**

*The values in bold represent significant changes during the period, as determined by the hypothesis test.

In 2023, a significant reduction in the average number of missing teeth was observed compared to 2010, across Brazil, its regions, and all analyzed subgroups. The largest reductions were among white individuals, with a decrease of 6.43 teeth (25.71%), followed by black individuals (4.74 teeth, 18.44%) and mixed-race individuals (4.46 teeth, 17.44%). Educational inequalities were also identified, with greater reductions in those with more years of schooling. The reduction in missing teeth was 6.55 teeth (37.36%) for elderly individuals with 12 or more years of schooling and decreased to 3.29 teeth (11.9%) for those with no schooling. Additionally, an increase in the number of filled teeth without caries was noted since 2003, while the number of untreated decayed teeth remained stable between 2023 and 2003. This increase in filled teeth without caries was observed in all regions, age groups, sexes, and self-reported race/skin color groups. Regarding education, there was a significant increase of filled teeth from 2010 compared to 2003 in individuals with higher educational levels, with no change from 2010 to 2023 (Table 2, Figura 2A, Figura 2B). Figure 2A shows lower averages of decayed and missing teeth and higher averages of filled teeth without caries among white individuals. Elderly individuals with higher educational levels showed lower DMFT averages, fewer missing teeth, and higher averages of filled teeth without caries (Figure 2B).

**Table 2:** Mean number of decayed, missing, and filled teeth in 2003, 2010, and 2023 for Brazil, regions, and subgroups according to sex, age group, and self-reported race/skin color.

Variables	Decayed			Missing			Filled		
	2003	2010	2023	2003	2010	2023	2003	2010	2023
Brazil	1.10 (1.01; 1.20)^a^	0.63 (0.52; 0.73)^b^	0.92 (0.73; 1.10)^a^	25.59 (24.98; 26.18)^b^	25.29 (24.66; 25.93)^b^	19.86 (18.88; 20.84)^a^	0.91 (0.77; 1.06)^c^	1.62 (1.38; 1.86)^b^	2.78 (2.28; 3.27)^a^
Region									
North	1.61 (1.30; 1.91)^b^	0.91 (0.74; 1.09)^a^	1.01 (0.77; 1.25)^a^	26.89 (26.10; 27.68)^b^	26.81 (26.18; 27.44)^b^	22.38 (20.86; 23.91)^a^	0.11 (0.06; 0.16)^c^	0.54 (0.30; 0.77)^b^	0.90 (0.65; 1.14)^a^
Northeast	1.93 (1.81; 2.05)^b^	0.93 (0.83; 1.03)^a^	1.11 (0.912; 1.31)^a^	24.87 (24.59; 25.15)^b^	25.18 (24.76; 22.60)^b^	21.41 (20.29; 22.53)^a^	0.31 (0.97; 1.20)^c^	1.08 (0.97; 1.20)^b^	1.39 (1.13; 1.65)^a^
Southeast	0.59 (0.46; 0.74)^bc^	0.53 (0.37; 0.69)^b^	0.99 (0.61; 1.36)^ac^	26.11 (24.88; 27.34)^b^	25.32 (24.34; 26.31)^b^	18.27 (16.40; 20.15)^a^	1.19 (0.87; 1.51)^c^	1.79 (1.43; 2.16)^b^	3.71 (2.74; 4.68)^a^
South	.960 (.875; 1.044)^b^	0.67 (0.51; 0.83)^a^	0.50 (0.40; 0.60)^a^	25.02 (24.56; 25.48)^b^	24.60 (23.66; 25.54)^b^	20.67 (19.35; 22.00)^a^	1.28 (1.17; 1.40)^c^	1.83 (1.49; 2.18)^b^	2.91 (3.35; 3.48)^a^
Central-West	1.29 (1.06; 1.52)^b^	0.78 (0.59; 0.97)^a^	0.69 (0.51; 0.86)^a^	25.14 (24.34; 25.95)^b^	25.66 (25.15; 26.17)^b^	21.31 (20.02; 22.60)^a^	1.13 (0.92; 1.34)^b^	1.05 (0.80; 1.30)^b^	2.45 (1.93; 2.97)^a^
Age (years)									
64–70	1.25 (1.15; 1.34)^c^	0.62 (0.53; 0.70)^b^	0.97 (0.73; 1.21)^a^	24.70 (24.22; 25.18)^b^	25.05 (24.29; 25.81)^b^	18.75 (17.60; 19.90)^a^	1.02 (0.84; 1.20)^c^	1.67 (1.38; 1.96)^b^	3.00 (2.49; 3.50)^a^
71–74	0.84 (0.64; 1.04)^a^	0.64 (0.42; 0.86)^a^	0.82 (0.65; 0.98)^a^	27.23 (25.94; 28.53)^c^	25.69 (24.92; 26.47)^b^	21.95 (20.85; 23.05)^a^	0.70 (032; 1.07)^c^	1.53 (1.20; 1.86)^b^	2.37 (2.79; 2.95)^a^
Sex									
Male	1.59 (1.45; 1.72)^c^	0.77 (0.55; 0.99)^b^	1.20 (0.95; 1.45)^a^	23.66 (23.16; 24.16)^b^	24.20 (23.28; 25.11)^b^	18.75 (17.54; 19.95)^a^	0.89 (0.77; 1.01)^c^	1.70 (1.30; 2.09)^b^	2.70 (2.24; 3.15)^a^
Female	0.73 (0.62; 0.84)^a^	0.54 (0.45; 0.63)^b^	0.73 (0.55; 0.91)^ab^	27.08 (26.29; 27.87)^b^	25.96 (25.22; 26.71)^b^	20.59 (19.47; 21.71)^a^	0.93 (0.69; 1.17)^c^	1.57 (1.34; 1.80)^b^	2.83 (2.22; 3.44)^a^
Race/Skin color									
White	0.76 (0.62; 0.90)^a^	0.54 (0.46; 0.62)^b^	0.83 (0.58; 1.09)^a^	25.46 (24.20; 26.72)^b^	25.01 (24.24; 25.78)^b^	18.58 (17.07; 20.09)^a^	1.24 (0.94; 1.55)^c^	2.03 (1.74; 2.32)^b^	3.69 (2.96; 4.42)^a^
Black	1.50 (1.27; 1.73)^a^	0.86 (0.34. 1.36)^b^	1.18 (0.86;1.50)^ab^	25.41 (24.12; 26.70)^b^	25.70 (24.21; 27.19)^b^	20.96 (19.55; 22.37)^a^	0.43 (0.13; 0.72)^b^	0.70 (0.55; 0.84)^b^	1.74 (1.20; 2.29)^a^
Mixed-race	1.55 (1.40; 1.71)^c^	0.67 (0.54; 0.79)^b^	0.91 (0.73; 1.08)^a^	25.77 (25.20; 26.34)^b^	25.58 (24.52; 26.64)^b^	21.12 (20.27; 21.98)^a^	0.51 (0.33; 0.69)^c^	1.34 (0.95; 1.74)^b^	1.94 (1.62; 2.27)^a^
Asian + Indigenous	1.05 (0.49; 1.61)^c^	0.71 (0.26; 1.16)^b^	1.67 (0.68; 2.65)^a^	26.10 (23.56; 28.67)^b^	25.82 (22.64; 29.00)^b^	20.03 (17.36; 22.71)^a^	1.04 (0.27; 1.82)^c^	0.83 (0.45; 1.21)^b^	2.60 (0.86; 4.33)^a^
Years of study									
Never been to school	1.64 (1.43; 1.84)^b^	0.82 (0.33; 1.32)^a^	0.88 (0.68; 1.08)^a^	25.79 (25.10; 26.50)^c^	27.57 (26.30; 28.83)^b^	24.28 (23.09; 25.48)^a^	0.32 (0.15; 0.48)^a^	0.37 (0.16; 0.59)^a^	0.48 (0.33; 0.64)^a^
1–4	0.89 (0.74; 1.05)^b^	0.63 (0.50; 0.76)^a^	0.72 (0.58; 0.86)^a^	26.65 (25.63; 27.66)^b^	26.11 (25.32; 26.90)^b^	22.79 (21.30; 24.28)^a^	0.80 (0.53; 1.07)^a^	1.18 (0.95; 1.42)^b^	1.87 (1.28; 2.47)^c^
5–8	0.81 (0.64; 0.97)^a^	0.55 (0.42; 0.68)^b^	1.01 (0.69; 1.34)^a^	23.67 (22.28; 25.07)^b^	24.99 (23.94; 26.04)^b^	20.99 (19.62; 22.36)^a^	1.59 (1.21; 1.97)^a^	1.85 (1.34; 2.34)^a^	2.17 (1.47; 2.86)^a^
9–11	0.65 (0.53; 0.76)^a^	0.46 (0.34; 0.59)^b^	1.04 (0.57; 1.50)^a^	23.07 (21.67; 24.46)^b^	23.02 (21.53; 24.51)^b^	18.25 (16.26; 20.24)^a^	2.02 (1.23; 2.81)^b^	2.79 (2.12; 3.46)^ab^	3.41 (2.88; 3.93)^a^
> 12	1.34 (0.59; 1.80)^a^	0.59 (0.39; 0.78)^b^	0.83 (0.63; 1.03)^ab^	19.64 (17.31; 21.97)^b^	17.53 (15.44; 19.62)^b^	10.98 (9.50; 12.45)^a^	3.87 (3.12; 4.62)^b^	5.75 (4.65; 6.86)^a^	6.69 (5.50; 7.87)^a^

*Identical letters in the horizontal direction indicate no statistical significance between the surveys, no change in the compared means.

**Figure 3:**
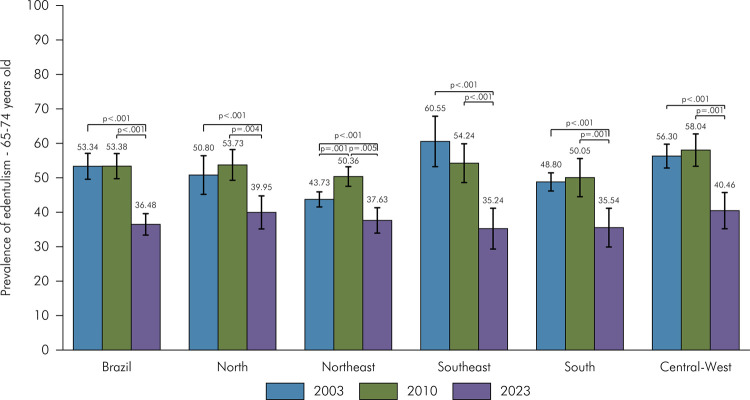
Prevalence of edentulism in Brazil and regions in 2003, 2010 and 2023.

The prevalence of edentulism was 53.34% in 2003, 53.38% in 2010, and 36.48% in 2023. A significant reduction was observed between 2023 and 2010, while the prevalence remained stable between 2003 and 2010. This reduction in 2023 was consistent across all Brazilian regions, age groups, sexes, and self-reported race/skin color groups (Figure 3, Table 3). A significant decrease in the prevalence of edentulism was also observed between 2023 and 2010 among elderly individuals with no schooling, as well as those with 1 to 4 and 5 to 8 years of schooling. On the other hand, among older people with 9 to 11 years and more than 12 years of schooling, although the 2023-point estimates were lower, the differences compared to 2010 were not statistically significant (Table 3).

p < 0.05 indicates statistically significant changes when comparing the three surveys.

**Table 3:** Prevalence of edentulism in 2003, 2010 and 2023 by subgroups according to sex, age group, and self-reported race/skin color.

Variables	2003	2010	2023	∆2010 – 2003	∆2023-2003	∆2023-2010
Age						
64– 70	47.82 (45.23; 50.40)	51.84 (47.35; 56.33)	31.70 (28.24; 35.16)	4.02 (-1.15; 9.20)	**-16.12 (-20.44; -11.79)**	**-20.14 (-25.81; -14.47)**
71–74	63.68 (55.15; 72.21)	56.01 (51.22; 60.79)	45.43 (41.18; 49.69)	-7.67 (-17.45; 2.11)	**-18.25 (-27.79; -8.71)**	**-10.57 (-16.97; -4.18)**
Sex						
Male	39.87 (36.92; 42.83)	49.56 (43.94; 55.18)	33.43 (29.88; 37.00)	**9.68 (3.34; 16.03)**	**-6.44 (-11.07; -1.81)**	**-16.13 (-22.78; -9.48)**
Female	63.83 (59.04; 68.62)	55.74 (51.66; 59.82)	38.47 (34.35; 42.60)	**-8.09 (-14.38; -1.80)**	**-25.36 (-31.68; -19.03)**	**-17.26 (-23.07; -11.46)**
Race/Skin Color						
White	55.43 (47.75; 63.11)	51.89 (47.75; 63.11)	34.50 (29.84; 39.13)	-3.54 (-12.32; 5.25)	**-20.94 (-29.92; -11.96)**	**-17.40 (-23.71; -11.10)**
Black	46.18 (38.96; 53.39)	56.53 (49.27; 63.80)	38.22 (32.33; 44.10)	**10.36 (0.12; 20.59)**	-7.96 (-17.27; 1.35)	**-18.32 (-27.66; -8.97)**
Mixed-race	51.71 (48.25; 55.16)	54.04 (48.95; 59.13)	38.71 (35.45; 41.98)	2.34 (-3.82; 8.49)	**-12.99 (-17.75; -8.24)**	**-15.33 (-21.37; -9.28)**
Asian + Indigenous	58.79 (41.65; 75.93)	62.97 (47.32; 78.61)	25.91 (10.61; 41.21)	4.17 (-19.03; 27.38)	**-32.88 ( -55.85; -9.91)**	**-37.05 (-58.94; -15.17)**
Years of study						
Never been to school	55.52 (51.97; 59.07)	65.70 (58.23; 73.16)	54.11 (48.87; 59.36)	**10.18 (1.91; 18.45)**	-1.41 (-7.75; 4.94)	**-11.58 (-20.71; -2.46)**
1–4	58.69 (51.59; 65.78)	57.74 (51.59; 65.78)	45.01 (39.53; 50.50)	-0.95 (-9.58; 7.69)	**-13.67 (-22.64; -4.70)**	**-12.72 (-20.09; -5.36)**
5–8	40.34 (32.65; 48.04)	51.54 (46.14; 56.95)	38.03 (31.89; 44.16)	11.20 (1.80; 20.61)	-2.32 (-12.16; 7.53)	**-13.52 (-21.69; -5.33)**
9–11	41.64 (35.81; 47.46)	35.62 (27.46; 43.77)	28.50 (22.99; 34.02)	-6.02 (-16.04; 3.99)	**-13.13 (-21.15; -5.11)**	-7.11 (-16.95; 2.73)
> 12 years	21.81 (20.29; 33.32)	21.23 (10.67; 31.80)	13.27 (9.82; 16.72)	-0.57 (-16.20; 15.06)	-8.53 (-20.55; 3.49)	-7.96 (-19.07; 3.15)

*The values in bold represent significant changes during the period, as determined by the hypothesis test.

**Figure 4:**
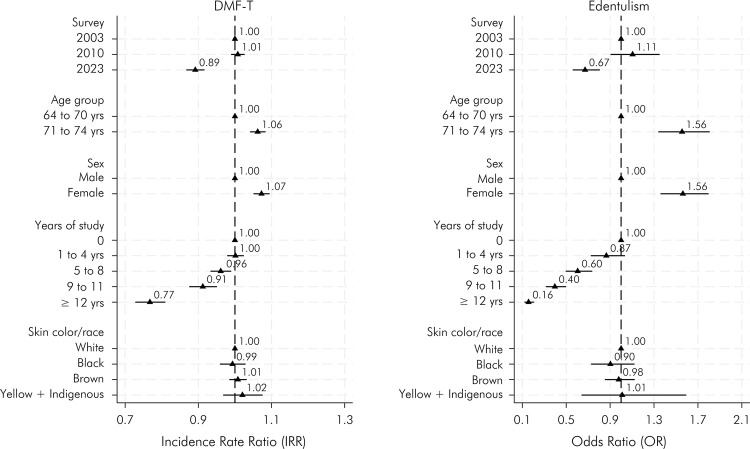
Association of DMFT and edentulism with survey year adjusted for sociodemographic characteristics of elderly individuals.

The regression model indicated a significant reduction in the DMFT (IRR: 0.89; 95% CI: 0.87; 0.92) and edentulism (OR: 0.67; 95% CI: 0.56; 0.81) between 2023 and 2003 after controlling for age, sex, self-reported race/skin color, and years of schooling. For both outcomes, there was a significant inverse association with years of schooling, i.e. higher educational levels were associated with lower DMFT and lower prevalence of edentulism (Figure 4). A significant reduction in the number of missing teeth was observed between 2023 and 2003, a result not observed between 2010 and 2003. There was also a significant increase in the number of filled teeth, a change also observed in 2010. A significant reduction in the number of decayed teeth was observed in 2010, but this was not observed when comparing 2023 and 2003. Elderly individuals with higher educational levels had fewer missing teeth and more filled teeth. Individuals who self-identified as mixed-race had a higher number of missing and decayed teeth and fewer filled teeth. Black individuals also had a significantly higher number of decayed teeth and fewer filled teeth.

## Discussion

The elderly Brazilian population is retaining more natural teeth, as evidenced by the reduction in the average number of missing teeth and the prevalence of edentulism in 2023 compared to 2010 and 2003. However, the observed variations occurred unevenly, being more pronounced among white individuals and those with higher levels of education, reflecting persistent inequities among different population subgroups.

Although significant reductions in the number of missing teeth and the prevalence of edentulism were observed in 2023 compared with previous surveys, tooth loss remains a problem among older people in Brazil. Approximately 35% of elderly individuals have edentulism, a prevalence higher than the estimated global average of 22.7% for individuals aged 60 or older.^7^ In 2010, the number of individuals aged 60 or older was 20,534,832, while in 2023, this number increased to 32,113,490, about a 50% growth in this age group.^25^ This means that, based on the survey estimates, approximately 10.9 million and 11.7 million people were living without teeth, respectively. Despite a reduction of about 30 percentage points in the prevalence of edentulism, this decrease was inferior to the population growth of this age group in the last 10 years. These data reveal that many Brazilians still age with missing teeth. The reduction in tooth loss was also evidenced by the decrease in the average number of missing teeth between 2010 and 2023. This reduction was reflected in the decline of dental caries experience, measured by the DMFT index, with 5.43 fewer teeth being extracted during this period. This pattern of reduced tooth loss is consistent with previous reports of greater percentages of older adults retaining their natural teeth in some countries, such as the United States,^26,27^ China,^13^ England and Wales,^12^ and Norway.^28^ These changes have been attributed to the benefits of fluoride during adolescence or youth, more positive patient and practitioner attitudes toward preventive measures, advances in dental technology leading to more treatment options (e.g., the introduction of high-speed handpieces/drills), and regular use of dental services.^13,26,27^

In Brazil, the 2010 national survey already indicated a reduction in tooth loss among adults aged 35 to 44 years, with the average number of lost teeth decreasing from 13.5 in 2003 to 7.48 in 2010.^29^ This reduction was attributed to the cohort effect, improvements in socioeconomic conditions, and strengthening of the healthcare system, including water fluoridation and increased use of fluoride toothpaste, which expanded in the 1980s and 1990s. In 2023, this reduction trend continued, with an average of 3.45 lost teeth among adults (data not presented). However, individuals aged 65 to 74 years, who were evaluated in 2023 and born in the 1950s and 1960s, did not benefit from collective preventive measures during childhood and adolescence, suggesting that the cohort effect has not yet significantly impacted this group. However, it is expected that in the coming decades, younger generations will experience even greater reductions in tooth loss as they age.

**Table 4:** Association of number of missing, decayed and filled teeth with survey year adjusted for sociodemographic characteristics of elderly individuals.

Variables	Missing	Decayed	Filled
	IRR (95% CI)	IRR (95% CI)	IRR (95% CI)
Survey year			
2003	1	1	1
2010	1.01 (0.98; 1.04)	**0.62 (0.51; 0.75)**	**1.42 (1.16; 1.73)**
2023	**0.85 (0.81; 0.89)**	0.85 (0.71; 1.02)	**1.91 (1.52; 2.39)**
Age group			
64–70	1	1	1
71–74	**1.09 (1.06; 1.12)**	**0.83 (0.71; 0.97)**	0.89 (0.77; 1.01)
Sex			
Male	1	1	1
Female	1.11 (1.07; 1.14)	**0.56 (0.50; 0.63)**	1.02 (0.88; 1.19)
Years of study			
Did not study	1	1	1
1–4	0.99 (0.96; 1.02)	**0.70 (0.60; 0.83)**	**2.98 (2.12; 4.17)**
5– 8	**0.92 (0.88; 0.97)**	0.80 (0.65; 1.00)	**4.02 (2.89; 5.59)**
9– 11	**0.82 (0.77; 0.88)**	0.83 (0.59; 1.17)	**6.00 (4.44; 8.10)**
≥ 12	**0.53 (0.48; 0.59)**	0.80 (0.62; 1.04)	**11.12 (8.22; 15.01)**
Self-reported race/skin color			
White	1	1	1
Black	1.02 (0.97; 1.07)	**1.57 (1.27; 1.93)**	**0.56 (0.46; 0.69)**
Mixed-race	**1.04(1.01; 1.08)**	**1.33 (1.11; 1.61)**	**0.65 (0.55; 0.76)**
Indigenous	1.03 (0.97; 1.10)	1.55 (0.96; 2.48)	0.76 (0.50; 1.18)

*The values in bold represent statistical significant associations.

The shift in dental practice from an interventionist to a preventive paradigm may have contributed to reducing tooth loss. Similarly, the reorientation of the dental practice of the oral health care model in Brazil, with the expansion of promotional, preventive, and conservative actions promoted by the National Oral Health Policy implemented in 2004, may have helped reduce extractions and preserve teeth with caries experience. These changes sought to overcome the historical mutilative pattern and restricted access to emergency treatments for adults and older people, contributing to the tooth loss scenario in the Brazilian elderly population.^30^ In fact, studies that analyzed procedures offered by SUS consistently observed a reduction in the rate of extractions compared to other procedures provided in primary care from 1998 to 2012^31^ and in extraction rates from 2008 to 2018.^32^ The increase in the number of filled teeth further supports the hypothesis that the shift in the oral health care model has had an impact, emphasizing the preservation of teeth with a history of caries through more conservative procedures. However, these changes in individual dental practice have not been sufficient to overcome health inequities.^33^

Despite the improvements, significant inequalities persist. The reduction in the prevalence of edentulism and in the number of lost teeth was more pronounced among white individuals and those with higher levels of education. On the other hand, worse indicators were concentrated among people with a low level of education and those who self-identified as mixed-race or black. Mixed-race individuals had a higher prevalence of untreated carious teeth and a lower number of filled teeth compared to white individuals. The results support previous studies that show an inverse relationship between level of education and oral health, with worse indicators concentrated among individuals in social vulnerability.^14,15^ In the elderly population, these inequalities reflect accumulated disease experiences over the life course and unequal access to healthcare services. Education, in this context, indicates the disparities experienced throughout life in employment opportunities, living conditions, access to healthy food, and access to oral health services. Individuals exposed to multiple adverse social determinants are at higher risk of negative outcomes, including caries and periodontal disease, the leading causes of tooth loss.^34-36^ The inequalities by race/skin color are a result of structural racism, which determines limited access to education, income, and healthcare services.^37^ Inequities in access to dental services for the black population may stem from barriers to dental services, racial discrimination in dental practice, and the historical neglect of the needs of the black population.^38^ Greater tooth loss may also result from the recommendation of cheaper and simpler treatments for black patients compared to white patients.^39^

Despite these inequalities, the increase in the number of filled teeth across all racial/skin color groups and among those with up to four years of schooling suggests that more vulnerable segments have, to some extent, benefited from expanding oral health services in SUS. This hypothesis also explains the reduction in the prevalence of edentulism across all racial/skin color groups and among individuals with low education levels. This hypothesis is further supported by the fact that the use of public oral health services is higher among mixed-race and black individuals compared to white individuals, as well as among those with lower levels of education, as demonstrated in the 2019 National Health Survey.^40^

This study used primary data from three national surveys to compare changes of oral health indicators among older people, a rapidly growing group in the population. Standardizing diagnostic criteria for dental caries across the three surveys enabled consistent comparison of changes in estimates over time. Additionally, adherence to WHO methodological guidelines regarding examiner training and calibration contributed to the validity of the results. The estimates obtained in the three surveys show good precision due to the large samples used. However, the surveys used samples collected at different time points, and the estimates should be interpreted in light of potential changes in social, economic, and public policy contexts over the studied period, which may have influenced oral health indicators. Population aging and changes in sociodemographic composition (such as education levels or access to healthcare services) may have impacted the results, making it challenging to separate demographic effects from variations in oral health indicators. Differences in sample composition regarding specific subgroups could introduce selection bias, affecting comparability.

The results reinforce the need to strengthen health promotion actions to preserve natural teeth, especially for populations of greater social vulnerability. Policies should integrate oral health approaches with strategies for other non-communicable diseases rather than continuing to treat oral diseases in isolation. These policies should adopt the principle of equity in the organization and provision of services and consider the material and social conditions that perpetuate inequalities through intersectoral actions.

## Conclusion

Edentulism and tooth loss have decreased among older adults aged 65 to 74 in Brazil. However, racial and educational disparities indicate that inequalities persist.
